# *Psidium guajava* and *Piper betle* Leaf Extracts Prolong Vase Life of Cut Carnation (*Dianthus caryophyllus*) Flowers

**DOI:** 10.1100/2012/102805

**Published:** 2012-04-29

**Authors:** M. M. Rahman, S. H. Ahmad, K. S. Lgu

**Affiliations:** Department of Crop Science, Faculty of Agriculture, Universiti Putra Malaysia, Selangor, 43400 Serdang, Malaysia

## Abstract

The effect of leaf extracts of *Psidium guajava* and *Piper betle* on prolonging vase life of cut carnation flowers was studied. “Carola” and “Pallas Orange” carnation flowers, at bud stage, were pulsed 24 hours with a floral preservative. Then, flowers were placed in a vase solution containing sprite and a “germicide” (leaf extracts of *P. guajava* and *P. betle*, 8-HQC, or a copper coin). Flowers treated with 8-HQC, copper coin, and leaf extracts had longer vase life, larger flower diameter, and higher rate of water uptake compared to control (tap water). The leaf extracts of *P. guajava* and *P. betle* showed highest antibacterial and antifungal activities compared to the other treatments. Both showed similar effects on flower quality as the synthetic germicide, 8-HQC. Therefore, these extracts are likely natural germicides to prolong vase life of cut flowers.

## 1. Introduction

The prospects for the cut flower industry in Malaysia are very promising due to the increase in the household and overseas markets. Per capita consumption of cut flowers per household had increased from 0.71 MYR in 1990 to 2.50 MYR in 2010. This trend is expected to increase further with greater consumer prosperity and aesthetic values for fresh cut flowers. There is also an increasing demand for cut flowers in the overseas market. This is expected to continue at a growth rate of 6% per annum. This value has reached 20 MYR and 36 MYR billion in the years 2000 and 2010, respectively.

Normally, dealers and consumers in cut flowers will put on view or store cut flowers in vases containing tap water plus sugar as the preservative or vase solution. When the bud is cut off from the mother plant, the bud no longer obtains its food from the mother plant. Sugar is used to provide food and more energy for bud opening and further flower development. Most preservative solutions contain two basic components: sugar and germicide. The sugar provides a respiratory substrate, whereas the germicide controls harmful bacteria and prevents plugging of the conducting tissues. The germicidal quality of 8-hydroxyquinoline citrate (8-HQC) ensures that bacteria do not grow in the vase solutions which may result in a blockage of vascular tissues and inhibition of water uptake by the flowers. According to Jones and Truett [1], germicides extended the vase life of *Gloriosa rothschildiana *mainly by improving solution uptake. The extracts from mature leaves of *Psidium guajava *and* Piper betle *have antimicrobial properties [2]. The leaf extracts of* P*. *guajava* contain phytochemicals, which act as antimicrobial, astringent, and bactericide, while the leaf extract of* P*. *betle* has antifungal, antiseptic, and anthelmintic activity. The high levels of five propenylphenols (chavicol, chavibetol, allylpyrocatechol, chavibetol acetate, and allylpyrocatechol diacetate) in *P*. *betle *leaf extracts showed favourable response towards fungicidal and nematocidal activity.

A major form of deterioration in cut flowers is the blockage of xylem vessels by air and microorganisms that cause xylem occlusion [3]. The 8-HQS is a tremendously beneficial germicide in preservatives used in the floral industry [4] and acts as an antimicrobial agent [5] which can increase water uptake [6]. The application of 8-HQS increased the vase life as well as the fresh weight (percentage of initial) of the cut flowers. The 8-HQS treatment also prevented growth of microorganisms in xylem vessels of the cut flower stems and maintained water uptake. The combination treatment of 8-HQS and sucrose improved the postharvest quality of gladiolus spikes [7]. In *Dendrobium* cut flowers, holding solutions containing 8-HQS + sucrose extended the vase life and improved flower quality, water consumption, fresh weight, flower freshness, and reduced respiration rate and weight loss [8].

Synthetic germicides such as silver thiosulphate (STS), silver nitrate (AgNO_3_), and 8-hydroxyquinoline citric (HQC) are expensive and not easily available in the local market. Furthermore, synthetic germicides, which contained silver, can pollute the environment due to its high phytotoxicity potential with harmful heavy-metal environmental contaminant [9]. The objectives of this research were to evaluate the effects of leaf extracts of *P*. *guajava* and *P*. *betle *on prolonging the vase life of cut carnation (*Dianthus caryophyllus*) flowers by controlling the microbial growth in the vase solution.

## 2. Materials and Methods

The experiment was conducted in the Postharvest Laboratory, Department of Crop Science, Universiti Putra Malaysia (UPM). The opened-bud stages of “Carola” and “Pallas Orange” carnation cut flowers were obtained directly from a supplier in the Cameron Highlands, Pahang. Cut carnations with firm buds and leaves that were free from damage, decay, and pest and diseases were selected. Each flower stalk was recut to a length of 45 cm. Leaves from the lower 15 cm of the stems were removed to avoid contamination in the vase solution. Then, the flower stalks were given pretreatments of sugar by submerging 10 cm of the stem ends in a vase solution containing 10% sucrose, 250 mg/L citric acid, and 250 mg/L 8-HQC for 12 h.

A 250 mL bottle was lined with a plastic tube (baby bottle system). This is to prevent contamination between tests and avoid the need for bottle washing. Sprite, a sterile-carbonated drink of pH 3.5 containing 26 grams of total sugar, 43 Kilocalories (Kcal) of energy, 3% citric acid, was used as the main vase solution into which either 8-HQC (250 mg/L), one copper coin, or leaf extracts (2 mL/L) of *P. guajava* or *P. betle* was added. Then, the pulsed flower stalk was divided randomly and placed into each tube, leaving the basal end about 1 cm away from the base of the bottle.

The flowers were evaluated at room temperature (25 ± 2°C) under continuous white fluorescent light at 1.2 klux. Vase life (day), flower diameter (cm), water uptake (mL), and petal colour were measured every other day. Vase life was measured as the longevity of flowers in the vase solution. Vase life was ended when the flower heads started to slump, followed by discolouration and abscission of petals, or when 30% of general appearance is no longer attractive. Flower diameter opening was measured by taking two measurements, which crossed at the centre of the flower, and the mean was calculated. Water uptake was measured daily by weighing the vase without the flower:

Water uptake (mL) on day *N* = Total weight (g) on day *N* − Total weight (g) on day *N*_−1_.
Rate of water uptake (mL) = Amount of water taken up (mL/day).
Total water uptake = Water uptake on day *N* + *N*_+1_ + *N*_+2_ ⋯ *N*_+*n*_.

Petal colour was measured on an alternate day for 9 days. The measurement was made using a chromometer (Model CR-300b, Minolta, Ramsey, NJ, USA) in the lightness (L*), chromaticity (C*), and hue (*h*°) colour notation system (C illuminant, calibrated with standard white plate and 0° viewing angle).

### 2.1. Microbial Growth

The evaluation of microbes was conducted to determine the effectiveness of the leaf extracts of *P. guajava* and *P. betle* in controlling the microbial growth in the vase solution of the cut carnation flowers. Fourteen gram of ready mixed nutrient agar (NA) and 20 g of potato dextrose agar (PDA) was each diluted in 500 mL of distilled water. These two liquid media were sterilized at 121°C at 15 psi for 15 minutes in an autoclave. Then, 20 mL of each medium was poured separately into a 100 mm sterile petri dish and cooled for a few hours to 30°C under a laminar flow. The flowers were left in the vase solutions for 6 days. Then, by using an Eppendorf micropipette, 100 *μ*L of the vase solution was taken from each treatment. Using an inoculation loop, a drop of the vase solution was taken and struck onto each of the NA and PDA media. Then, the petri dishes were incubated at 30°C in an incubator for 24 and 72 h, for bacterial and fungal growth, respectively. All the plates were evaluated for microbial growth. Then, growth of each microorganism was evaluated based on scores of 0–5: None = 0 colony/plate, Few = 1-2 (<30 colonies/plate), Moderate = 3-4 (30–300 colonies/plate), and Abundant = 5 (>300 colonies/plate) as shown in Table 1.

### 2.2. Leaf Extracts of *P. guajava* and *P. betle*

Leaves were extracted according to the methods of Rahman et al. [10] with some modifications. Fresh and mature leaves of *P. guajava* and *P. betle *were collected and sliced into small pieces (0.5 cm × 3 cm). The sliced leaves were dried at room temperature (27 ± 2°C) for 3 to 4 days and then grounded into a powder form. Then, the leaf was macerated with 80% ethanol for 24 hours at room temperature. Each extract was filtered and then evaporated with a rotary evaporator (Model CA-1310 Eyela, Tokyo Rikakikai Co., Ltd., Japan) under reduced pressure at a temperature not exceeding 50°C. The dark brown viscous residue was reconstituted in ethanol (5 mL). Two millilitre of each extract was mixed into 1 L of sprite to act as the vase solution.

## 3. Results

### 3.1. Flower Diameter

**Figure 1:**
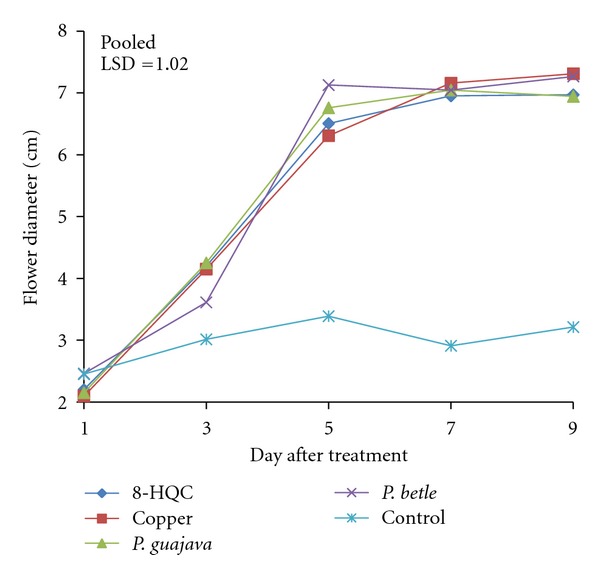
Effect of 8-HQC, copper coin, and leaf extracts of *P. guajava* and *P. betle *and control on flower diameter of “Carola” cut carnation flowers.

The Carola carnation treated with 8-HQC, copper coin, and the leaf extracts of *P. guajava* and *P. betle *showed a similar trend in flower opening as indicated by the increase in flower diameter (Figure 1). All flowers were partially opened on day 3. From days 3–5, there were rapid increases in diameter of flowers treated with 8-HQC, copper coin, and leaf extracts of *P. guajava *and *P. betle*. On day 5, control flowers had already attained the maximum diameter opening. Similarly, flowers treated with 8-HQC, copper coin, leaf extracts of *P. guajava *and *P. betle *obtained maximum diameter opening on day 5 and the diameter opening level off until day 9, by which time the flowers began to senesce. Similar results were found for the Pallas Orange flowers (data not included). After 9 days, flowers treated in tap water lost their turgidity and started to turn black and wilt.

On day 5, flowers treated with leaf extract of *P. betle* had significantly larger flower diameter compared to flowers treated with 8-HQC and copper coin (Figure 1). By day 9, diameters of flowers treated with 8-HQC, copper coin, and leaf extracts of *P. guajava *and *P. betle *were not significantly different from one another. The control flowers had the smallest diameter compared to flowers in all the other four treatments, throughout the evaluation period of the flowers. The cloudiness of the control vase solution (tap water) indicated that there was an abundance of microbes. These microbes clogged the xylem vessels of the flower stem.

### 3.2. Water Uptake

**Figure 2:**
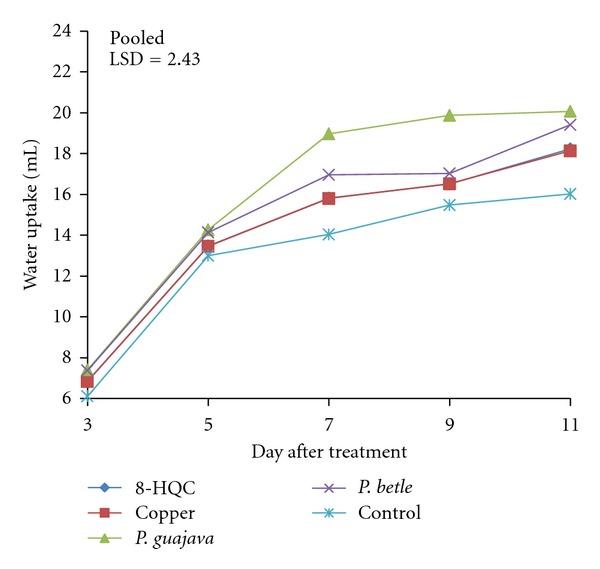
Effect of 8-HQC, copper coin, and leaf extracts of *P. guajava* and *P. betle* and control on water uptake of “Carola” cut carnation flowers.

There were no significant differences in water uptake of the Carola carnation when treated with leaf extracts of *P. guajava* and *P. betle*, copper coin, 8-HQC, and control from days 3–5 after treatment (Figure 2). However, flowers treated with 8-HQC, copper coin, and *P. Betle *leaf extracts had no significant differences in water uptake throughout the study. On days 7–9, flowers treated with leaf extracts of *P. guajava *had significantly higher water uptake compared to the other treated flowers. But by day 11, flowers in all the four treatments were not significantly different in their water uptake. Water uptake of the control flowers was at the lowest compared to all the four treated flowers on day 11, due to flower senescence. However, water was still taken up for leaf survival.

The flowers treated with copper coin, 8-HQC, and leaf extracts of *P. guajava *and *P. betle* had gradual increases in water uptake from days 5–11. Similar results were found for Pallas Orange carnation flowers (data not included). Throughout the period of the evaluation, it was observed that water uptake of the control flowers were lowest after days 9–11 compared to flowers in leaf extracts of *P. guajava* and *P. betle*. In general, flowers treated with leaf extracts of *P. guajava *and *P. betle *had the higher water uptake compared to the control. Both 8-HQC and copper coin had a similar trend of water uptake from days 3–11.

### 3.3. Vase Life

There was no significant difference in the vase life of Carola carnation treated with copper coin and leaf extracts of *P. guajava* and *P. betle* throughout the evaluation period (Table 2). This showed that both of the leaf extracts could prolong the vase life of the flowers. Similarly, vase life of the Pallas Orange carnation treated with copper coin and leaf extracts of *P. guajava* and *P. betle* were significantly different from the control flowers (Table 2). Both types of flowers placed in the vase solution containing either 8-HQC, copper coin, or leaf extracts of *P. guajava* and *P. betle *had 5–7 days longer vase life compared to control flowers. In general, the vase life of flowers was terminated when 30% of the petals were rolled in and had senesced.

### 3.4. Petal Colour

**Figure 3:**
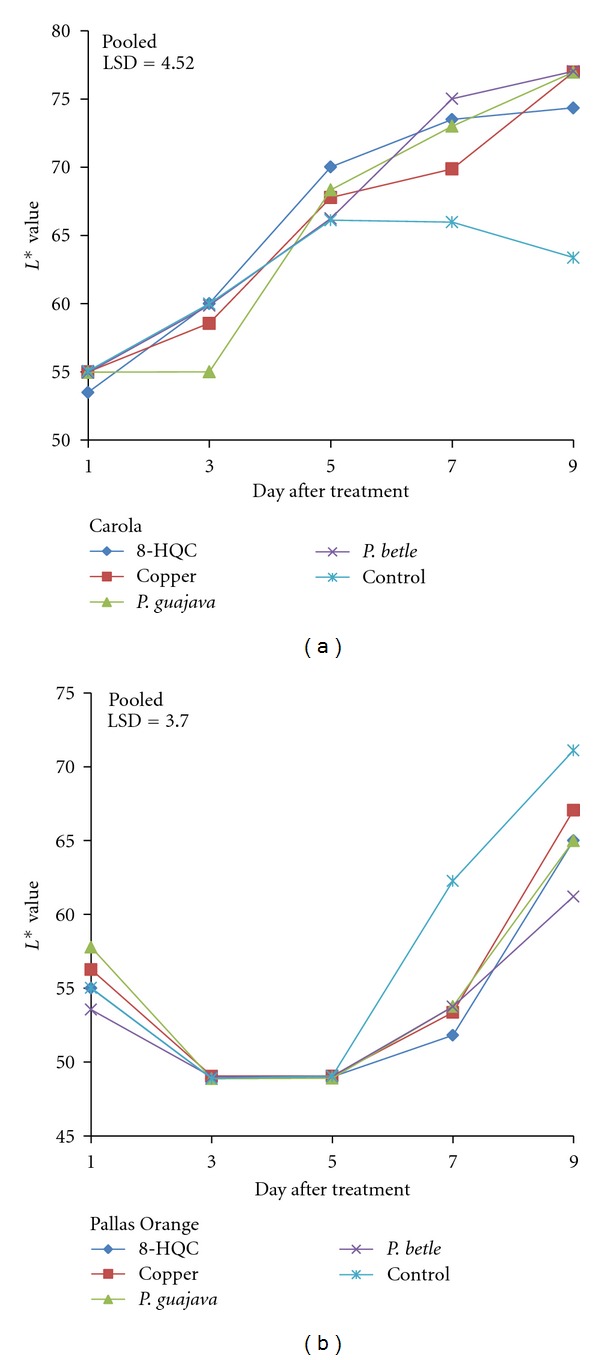
Effect of 8-HQC, copper coin, and leaf extracts of *P. guajava* and *P. betle* and control on L* value of (a) “Carola” and (b) “Pallas Orange” cut carnation flowers.

Larger L* values indicate a lighter colour compared to smaller L* values that indicate a darker colour (0 = black to 100 = white). The L* values of pink Carola flowers in all the treatments increased from days 1–9 except for flowers in the control treatment, which decreased after day 5 (Figure 3(a)). The L* value of control flowers decreased until day 9. For the treated flowers, L* value continued to increase, indicating that the flowers had become brighter in colour. However, all the Pallas Orange carnation flowers showed a decreasing trend of L* values from days 1–5 (Figure 3(b)). From days 5–9, the L* for the control flowers showed a rapid increase and was significantly different from the treated flowers. This indicated that petal of the control flowers became lighter than those treated with 8-HQC, copper coin, and leaf extracts of *P. guajava *and *P. betle.*

**Figure 4:**
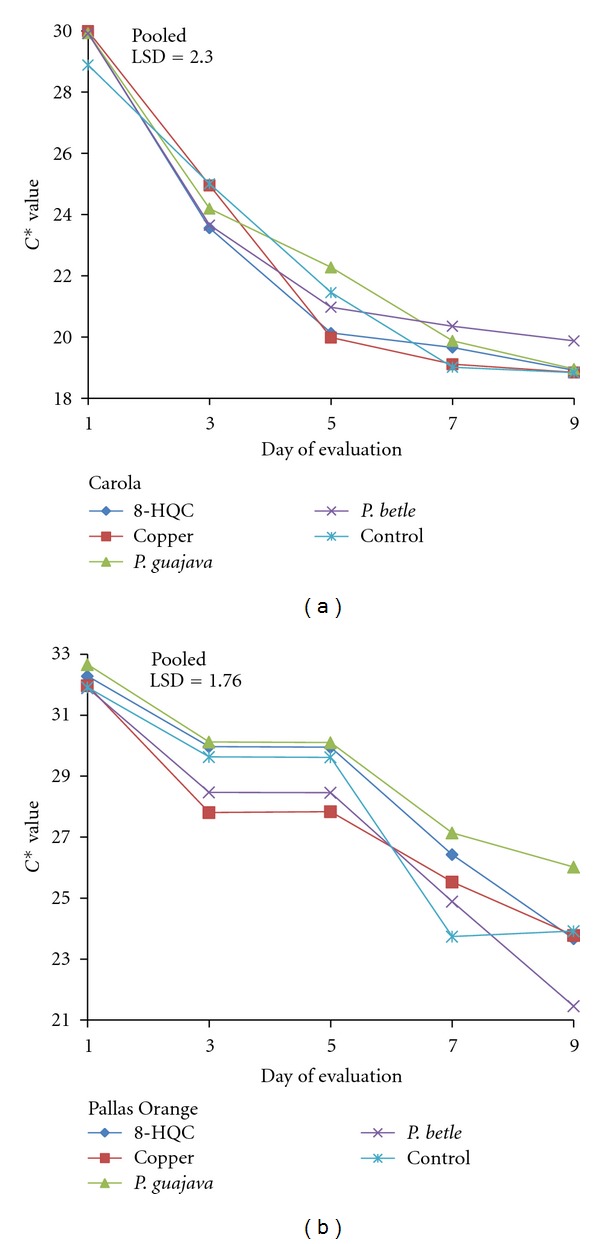
Effect of 8-HQC, copper coin, and leaf extracts of *P. guajava* and *P. betle* and control on C* value of (a) “Carola” and (b) “Pallas Orange” cut carnation flowers.

 There were initial rapid declines in C* values for Carola carnation in all the treatments from days 1–5 (Figure 4(a)) followed by a levelling off of C* values until day 9. These indicated the loss of vividness or saturation of colour as the flowers senesced. The Pallas Orange carnation flowers showed a similar trend of decrease in C* values throughout the evaluation period, except for days 3–5, during which flowers treated with *P. guajava *extract and copper coin were significantly different in C* values from flowers treated with leaf extract of* P. guajava*, 8-HQC, and control (Figure 4(b)). After day 5, flowers treated with 8-HQC, leaf extract of *P. betle,* and the control showed a decline in C* value. In general, by day 9, the flowers treated with leaf extract of *P. guajava* had the highest C* value, while flowers treated with leaf extract of *P. betle *had the lowest C* value.

**Figure 5:**
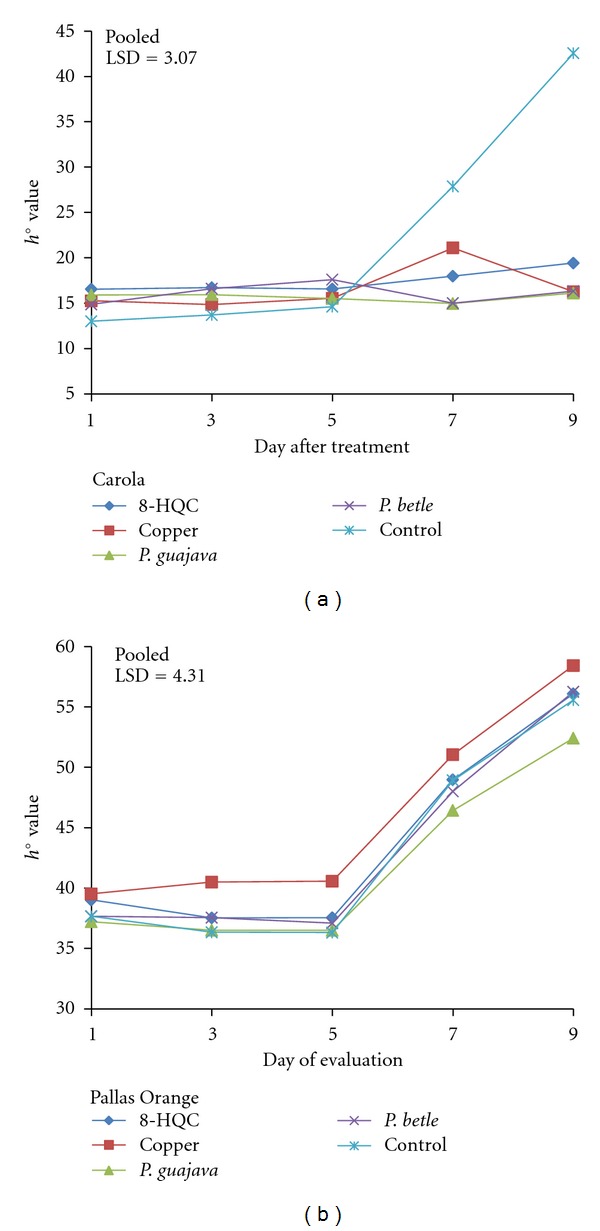
Effect of 8-HQC, copper coin, and leaf extracts of *P. guajava* and *P. betle* and control on *h*° value of (a) “Carola” and (b) “Pallas Orange” cut carnation flowers.

 From days 1–5 after treatment, Carola carnation flowers treated with 8-HQC, copper coin, and leaf extracts of *P. guajava* and *P. betle* had a similar trend of *h*° values throughout the evaluation period (Figure 5(a)). After day 5, the *h*° value of the control flowers showed a rapid increase indicating that the flowers had changed colour and senesce. From days 5–9, *h*° values of flowers treated with leaf extracts of *P. guajava* and *P. betle* and 8-HQC remained constant. Similarly, the colour of the Pallas Orange carnation flowers remained constant from days 1–5 (Figure 5(b)). After day 5, *h*° value increased rapidly indicating colour changes of flowers from orange to reddish orange. Flowers treated with the copper coin had significantly higher *h*° values from days 3–9 compared to flowers treated with leaf extracts of *P. guajava*.

### 3.5. Microbial Growth

**Figure 6:**
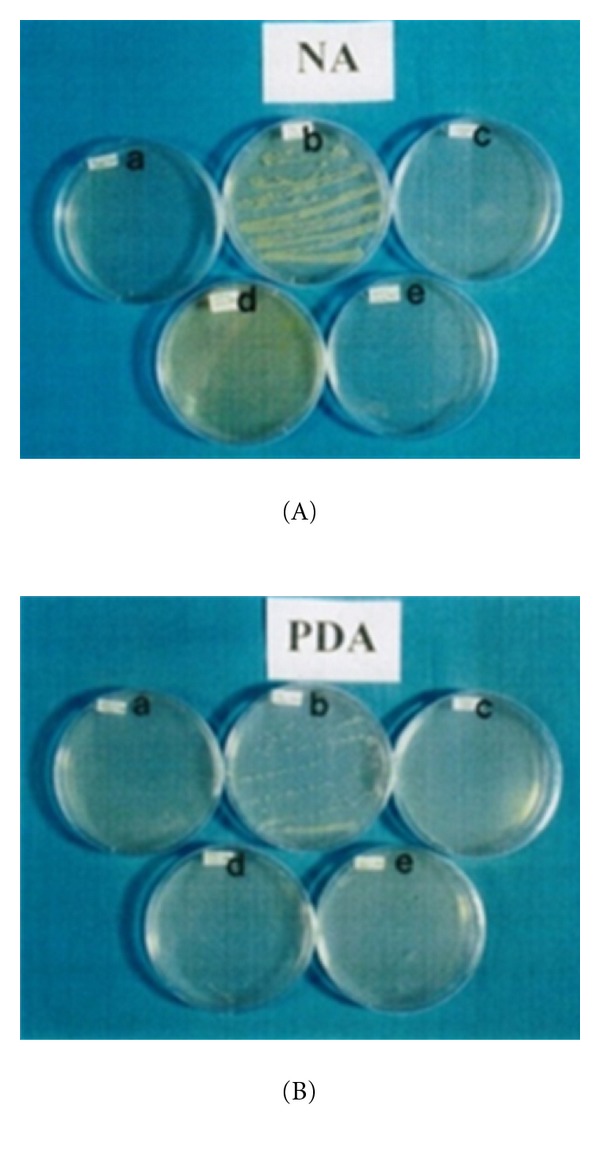
Growth of bacteria and fungi on (A) nutrient agar and (B) potato dextrose agar media plated on petri dish which contain copper coin (a), tap water (b), 8-HQC (c), *P. betle *leaf extract (d), and *P. guajava *leaf extract (e) on both media after day 2 of streaking vase solution of cut carnation flowers on day 9.

**Figure 7:**
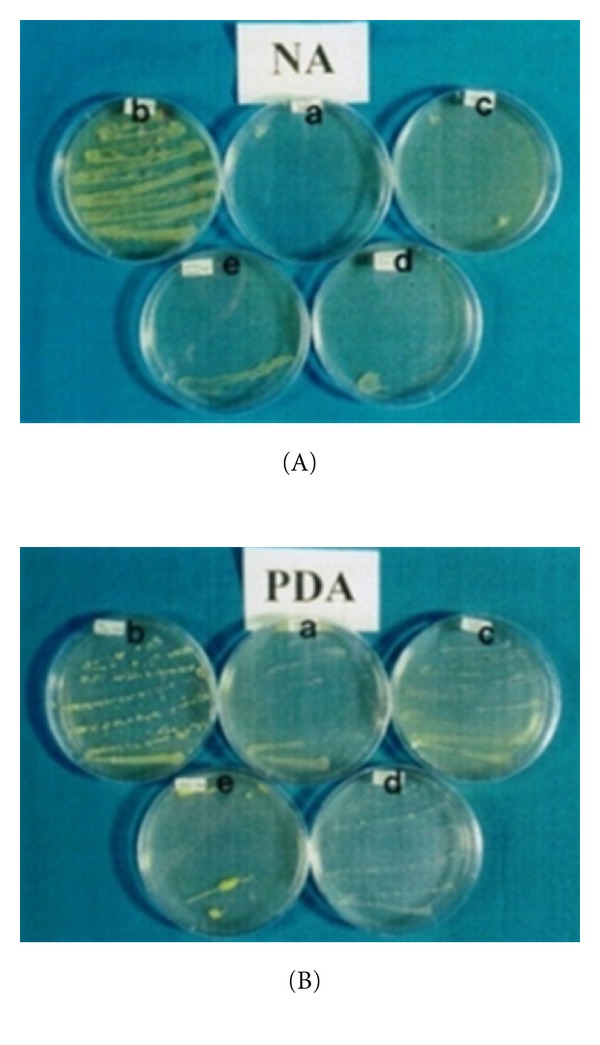
Growth of bacteria and fungi on (A) nutrient agar and (B) potato dextrose agar media plated on petri dish which contain copper coin (a), tap water (b), 8-HQC (c), *P. betle *leaf extract (d), and *P. guajava *leaf extract (e) on both media after day 3 of streaking vase solution of cut carnation flowers on day 9.

The control (tap water only) vase solution contained a significantly higher bacterial colony compared to the vase solutions containing 8-HQC, copper coin, and leaf extracts of *P. guajava* and *P. betle* on day 3 after streaking (Table 3). It was observed that the control solution had an abundant growth of bacteria which started on day 2 (Figure 6(A)). Similarly, on day 3 after streaking, leaf extracts of *P. betle* and *P. guajava*, copper coin, and 8-HQC showed the highest antibacterial activities as proven by a few bacterial colonies on the nutrient agar media (Figure 7(A)).

There were significant differences in the score of microbial growth on potato dextrose agar with the control vase solution compared with the vase solutions containing 8-HQC, copper coin, and leaf extracts of *P. guajava* and *P. betle *(Table 3). There was an abundant growth of fungi on the control solution. Leaf extracts of *P. guajava* showed the highest antifungal activities (Figure 7(B)). Other germicides like 8-HQC, copper coin, and leaf extract of *P. betle* did not appear to be as good antifungal germicides. All these microorganisms growth was evaluated based on a score of 0–5, where None = 0 colony/plate, Few = 1-2 (<30 colonies/plate), Moderate = 3-4 (30–300 colonies/plate), and Abundant = 5 (>300 colonies/plate).

## 4. Discussion

In the present study, treated flowers had almost 30% larger flower diameter opening compared to control. Similarly, cut lotus (*Nelumbo nucifera*) flowers fail to open, and petal blackening occurs due to carbohydrate depletion in the leaves and sink activity of the flowers [11, 12]. Thus, it is not practical to use tap water alone, without any food source, as a vase solution for carnation flowers. All holding solutions must contain essentially two components: sugar and germicides. The sugar provides a respiratory substrate, while the germicides control harmful bacteria and prevent plugging of the water-conducting tissues. Among all the different types of sugars, sucrose has been found to be the most commonly used sugar in prolonging vase life of cut flowers. Sucrose added to the vase solution supplies cut flowers with substrates that are needed for respiration, thus enabling harvested buds to open into flowers [13]. The water uptake of tap water depends on factors like acidity (pH), total dissolved solids, and the presence of specific toxic ions [14]. The most toxic ion in tap water to carnation flower development is fluoride, and flowers were injured by 1 ppm fluoride [15]. A similar observation was found where decline in water uptake in rose was caused by bacteria ranging from 10^7^ to 10^8^/mL in the vase solution [14]. At 3 × 10^9^/mL, the first sign of wilting would appear in an hour. Additionally, decreases in water uptake, which closely followed an increase in the tonoplast permeability, caused senescence in cut carnation flowers [16]. In the present study, initial increase of 20–30% water uptake rate indicated that water was taken up through the stems to supply adequate food and nutrition for the flower development. A rapid decline of water uptake for the control flowers after day 5 was associated with senescence and abscission of the older leaves or flowers in the final phase of development [17, 18]. Cloudiness of the vase solution due to the activities of microbes could contribute to early senescence of the flowers. A similar observation was found whereby carnation flowers only lasted for 5-6 days in tap water [19]. The reasons for the short vase life of the control flowers were associated with the presence of microbes in the vase solution. Metabolites produced by certain bacteria also reduced longevity and water conductivity in carnation [20].

Senescence in carnation flowers was mainly associated with sensitivity of the carnation flowers to ethylene and carbohydrate metabolism. Ethylene was produced during normal metabolism in flowers and conditions created by infection, wound, and stress, like wilting, which accelerated its biosynthesis [21]. Other factors like microbial infection and lack of food supply are very important in affecting vase life. Pretreatment with sucrose (10%) could prolong the vase life of cut flowers. Pulsing with concentrations of sucrose > 10% for 20 h prolonged the vase life of the spikes of cut *Liatris spicata *[22]. Furthermore, recutting stem ends could help in prolonging the vase life of cut flowers. Recutting the stem end promotes rehydration of the stems, speeds up flower opening, quickly revives wilted flowers, and increases the lasting qualities of the cut flowers [19]. Pretreatment with sugar improved colour and sizes of petals and the vase life of a number of cut flower species [23]. This requirement for sugar explained why the control flowers that were treated in tap water alone had poor colour. There were no significant differences in L* values among flowers treated with 8-HQC, copper coin, and leaf extracts of *P. guajava* and *P. betle*, except that the control in both types of cut carnation flowers Pallas Orange carnation had orange petals, while the Carola carnations had pink flowers. Initially on day 1, the Carola carnation flowers were light pink, with low L* and *h*° values but high C* values. From the result, it is apparent that the optimum aesthetic values of the flowers were between days 4 and 6. During this period, the diameter of the germicide-treated flowers were about 65–95% larger than control flowers. The larger flower diameter indicated a normal opening behavior of the flowers. The diameter opening of control flowers was not normal throughout the evaluation period. This abnormal opening resulted in lower L* and higher *h*° values of control flowers compared to the germicide-treated flowers, by day 9. As for the Pallas Orange carnation, the initial petals had low L* and *h*° but with high C* colour values. The results indicated that, by day 7, color saturation of control flowers had decreased, resulting in fading. Generally, when colour saturation of flowers decreased, the colour of the petals became darker, leading to a loss of flower colour. According to Salunkhe et al. [19], senescing flowers showed discoloration or fading of colour due to significant changes in carotenoid and anthocyanin pigments, which are responsible for different colours of flowers. NA of pH 7.4 and with a formulation was the favourite culture media for the growth of heterotrophic bacteria [24]. The control solution was full of bacteria cells since no germicide was used to control the microbial growth. Germicides controlled microbial growth and partially decreased the resistance to water uptake [14].

Hence, leaf extracts of *P. guajava* appeared to be an excellent, natural antimicrobial agent. It showed antibacterial activity against the bacteria tested, in the most cases, with activity stronger than 50 *μ*g streptomycin. Polyphenolic compounds like guaijaverin, quercetin, and avicularin are the active antimicrobial components in guava leaf [2]. Leaf extracts of *P. betle* contain two phenols, betel-phenol (chavibetol) and chavicol. Leaf extracts of *P. betle* had antifungal activities [2]. The high levels (1% of leaf fresh weight) of five propenylphenols (chavicol, chavibetol, allylpyrocatechol, chavibetol acetate, and allylpyrocatechol diacetate) were considered responsible for the fungicidal and nematocidal activities. Leaf extracts of *P. betle* have been shown to be effective against pathogens that cause collar rots, such as *Pyricularia oryzae* Cav., *Cochliobolus miyabeanus*, *Rhizoctonia solani* Kuhn, and *Thanatephorus cucumeris* (Frank) Donk [25].

## 5. Conclusions

It is not reasonable to use tap water alone as a vase solution to preserve carnation flowers. The response of Carola and Pallas Orange carnation flowers to tap water was a lower water uptake, shorter vase life, and smaller flower diameter compared to treated carnation flowers. In addition, tap water of pH 7.2 provided a favourable environment for bacteria and fungi to multiply in the vase solution.

Sprite, in combination with leaf extracts of *P. betle* or *P. guajava,* was able to prolong the vase life of Carola and Pallas Orange carnation flowers. The high concentrations of sugar, 10%, in the sprite had become the main source of energy for maintaining the biochemical and physiological processes after the flowers were separated from the mother plant. The presence of sodium benzoate and carbon dioxide in the sprite inhibited ethylene action while the leaf extracts of *P. guajava* and *P. betle* acted as germicides.

When treated with leaf extracts of *P. guajava* and *P. betle,* longevity of the Carola and Pallas Orange carnation flowers doubled when compared to the flowers placed in the control solution. These results indicated that leaf extracts of *P. guajava* and *P. betle *were able to prolong the vase life of carnations flowers. Conversely, carnation flowers that were pulsed overnight in high concentrations of sucrose (10%) together with 250 mg/L 8-HQC and 250 mg/L citric acid could extend longevity of the carnation flowers. However, longevity and colour of flowers in the vase solution depend much more on the variety of the carnation flowers. In this experiment, a slightly longer vase life and gradual decrease in colour saturation (C* values) were observed in the Pallas Orange than Carola carnation flowers.

Flowers treated with leaf extracts of the *P. guajava* and *P. betle* showed similar results in diameter opening, water uptake, and vase life as the other synthetic germicides like 8-HQC and copper coin. Therefore, they have potential as natural germicides for antibacterial activities. The leaf extracts of *P. guajava* and *P. betle *contain phenolic compounds, either bactericidal or bacteriostatic, depending on the concentration used. Chavicol and chavibetol are compounds found in *P. betle* leaf extracts. They are responsible for the antifungal activities. However, further investigation should be carried out to determine the actual concentration of leaf extracts of *P. guajava* and *P. betle* to inhibit the growth of bacteria and fungi in the vase solution.

## Figures and Tables

**Table 1:** Scheme for scoring growth of microorganism based on colony.

Microorganism growth	Colony	Score
Abundant	(>300 per plate)	5/5
Moderate	(30–300 per plate)	3/5
Few	(<30 per plate)	1/5
None	0	0

**Table 2:** Effect of 8-HQC, copper coin, and leaf extracts of *P. guajava* and *P. betle* and control on vase life of “Carola” and “Pallas Orange” cut carnation flowers.

Treatment	Vase life (days)	
	“Carola”	“Pallas Orange”
Sprite + 8-HQC	9.6 b^z^	10.6 b^z^
Sprite + copper coin	11.0 a	12.3 a
Sprite + *P. guajava *	10.3 ab	11.1 ab
Sprite + *P. betle *	10.4 ab	11.5 ab
Tap water (control)	4.8 c	5.1 c

^Z^Comparison of means within columns by LSD (*P* = 0.05). Means are averages for 8 replications with one flower stalk per replicate.

**Table 3:** Effect of vase solution containing 8-HQC, copper coin, leaf extracts of *P. guajava*, and *P. betle* and control at seven days after treatment on the microorganism growth after three days of inoculation. Media used were nutrient agar (NA) and potato dextrose agar (PDA).

Treatment	Score of microorganism growth	
	NA	PDA
Sprite + 8-HQC	1.4 b^z^	3.4 b^z^
Sprite + copper coin	1.0 b	2.4 c
Sprite + *P. guajava *	1.4 b	2.2 c
Sprite + *P. betle *	1.2 b	3.4 b
Tap water (control)	5.0 a	4.8 a

^Z^Comparison of means within columns by LSD (*P* = 0.05). Means are averages for 5 replications.
